# Proteolytic and Opportunistic Breaching of the Basement Membrane Zone by Immune Cells during Tumor Initiation

**DOI:** 10.1016/j.celrep.2019.05.029

**Published:** 2019-06-04

**Authors:** Maaike C.W. van den Berg, Lucy MacCarthy-Morrogh, Deborah Carter, Josephine Morris, Isabel Ribeiro Bravo, Yi Feng, Paul Martin

**Affiliations:** 1 School of Physiology, Pharmacology & Neuroscience, Biomedical Sciences Building, University of Bristol, University Walk, Bristol BS8 1TD, UK; 2 School of Biochemistry, Biomedical Sciences Building, University of Bristol, University Walk, Bristol BS8 1TD, UK; 3 University of Edinburgh Centre for Inflammation Research, Queen’s Medical Research Institute, Edinburgh BioQuarter, Edinburgh EH16 4TJ, UK; 4 School of Medicine, Cardiff University, Cardiff CF14 4XN, UK

**Keywords:** cancer, inflammation, zebrafish, CLEM, basement membrane zone, neutrophils, macrophages, cell motility, collagen

## Abstract

Cancer-related inflammation impacts significantly on cancer development and progression. From early stages, neutrophils and macrophages are drawn to pre-neoplastic cells in the epidermis, but before directly interacting, they must first breach the underlying extracellular matrix barrier layer that includes the basement membrane. Using several different skin cancer models and a collagen I-GFP transgenic zebrafish line, we have undertaken correlative light and electron microscopy (CLEM) to capture the moments when immune cells traverse the basement membrane. We show evidence both for active proteolytic burrowing and for the opportunistic use of pre-existing weak spots in the matrix layer. We show that these small holes, as well as much larger, cancer cell-generated or wound-triggered gaps in the matrix barrier, provide portals for immune cells to access cancer cells in the epidermis and thus are rate limiting in cancer progression.

## Highlights

• Macrophages and neutrophils breach the basement membrane zone to access cancer cells
• Breaching is by proteolysis or opportunistically through pre-existing weak spots
• Clones near BMZ weak spots gain a growth advantage from immune cell infiltration
• Wounds provide another portal for immune cells to gain access to the epithelium

## Graphical Abstract

**Figure:**
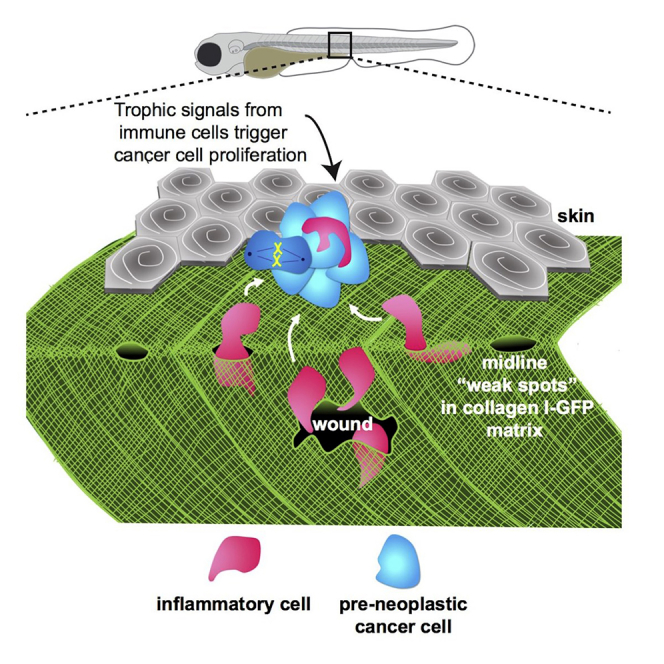

## Teaser

Van den Berg et al. use a zebrafish model to observe how inflammatory cells access early-stage pre-neoplastic cells in epithelia by breaching the extracellular matrix basement membrane zone. These breaches are opportunistic, often through pre-existing weak spots, and are rate limiting for cancer cell proliferation.

## Introduction

For any epithelial cancer to become malignant, it must breach the basement membrane extracellular matrix (ECM) barrier before commencing metastatic invasion. Defects in the basement membrane (BM) accompany local metastatic invasion of murine and human epithelial cancers (Chang et al., 2017, Frei, 1962, Glentis et al., 2017, Kinjo, 1978, Spaderna et al., 2006). However, from the earliest stages of cancer development, an inflammatory response is triggered by pre-neoplastic cells, and this can drive a proliferative response and trigger subsequent metastatic spread of the cancer (Chia et al., 2018, Coffelt et al., 2015, Feng et al., 2010, Feng et al., 2012, Freisinger and Huttenlocher, 2014, Kitamura et al., 2015a, Kitamura et al., 2015b). For inflammatory cells to directly interact with pre-neoplastic cells, they too must breach the basement membrane, but in the reverse direction, from the dermal connective tissue into the epidermis. Similar basement membrane breaching is seen during development (Sherwood and Sternberg, 2003) and also when immune cells diapedese through vessel walls (Voisin et al., 2010). The early stages of cancer initiation are difficult to live-image in the opaque tissues of mice and human. However, the translucent zebrafish larvae, in which both pre-neoplastic cells and immune cells can be fluorescently labeled, offer the possibility of visualizing the moments when basement membrane breaching by inflammatory cells occurs.

Here we use inducible models to generate HRAS^G12V^-expressing epidermal pre-neoplastic cells (Ramezani et al., 2015). This allows us to observe how one oncogene, mosaically expressed in specific cell lineages, can disrupt the local skin architecture and trigger an inflammatory response. We combine these models with a transgenic zebrafish line in which epidermal collagen Iα2 is fluorescently labeled to reveal a meshwork of ECM immediately beneath the basement membrane (Morris et al., 2018), which together we refer to as the basement membrane zone (BMZ) (Menter and Dubois, 2012, Nauroy et al., 2019). Using correlative light and electron microscopy (CLEM), we study precisely how immune cells traverse this barrier to access pre-neoplastic cells in the epidermis at these early cancer stages.

## Results and Discussion

### Cancer Initiation in Different Cell Lineages Causes Local Disruption of the Skin Architecture

**Figure 1:**
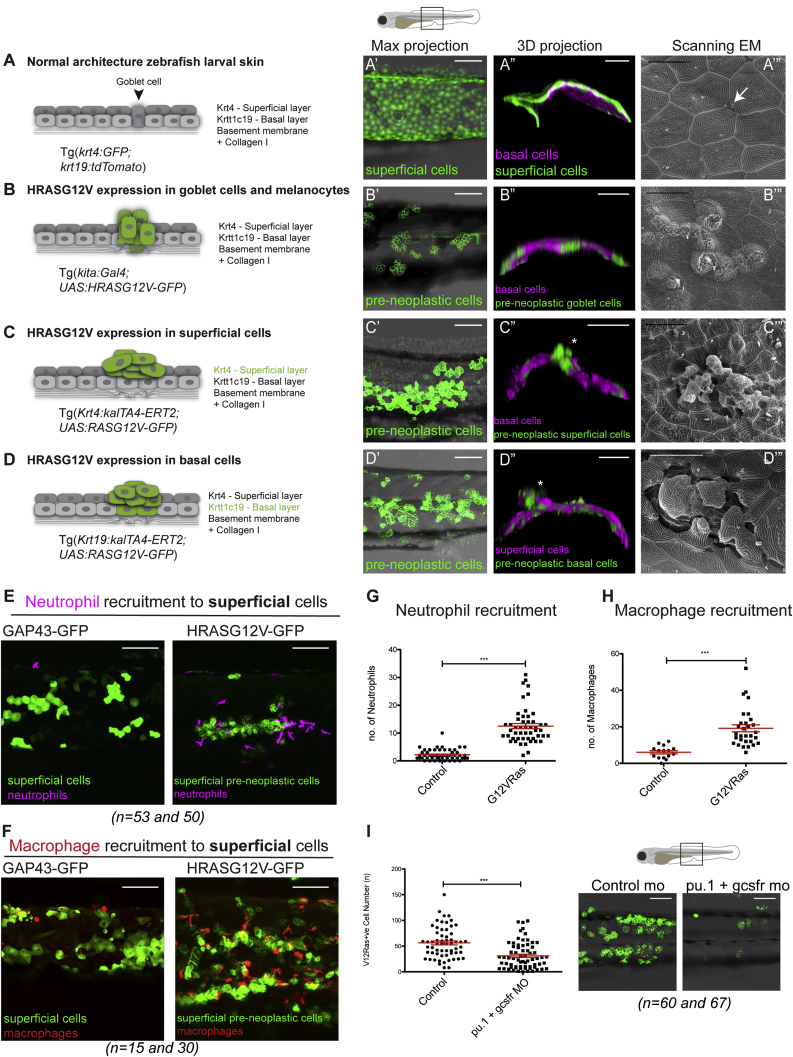
Zebrafish Skin Cancer Models and Immune Cell Recruitment (A) WT 3 dpf larval skin: superficial cell layer (dark gray in A, GFP [green] in A′ and A″) and basal cell layer (light gray in A, magenta in A″) with underlying basement membrane (BM). (A″′) Scanning electron microscopy shows a goblet cell (arrow in A and A″′, green in B) in the epidermis. (B) kita:RAS model. HRASG12V-GFP expressing goblet cells in 7 dpf larva over-proliferate (green in B, B′, and B″). Basal cells are in magenta (B″). Tracks of goblet cells by scanning electron microscopy (B″′). (C and D) K4:RAS and K19:RAS models. HRASG12V-GFP expression (48 h postinduction [hpi] of 4OHT) in superficial (C and C′) or basal (72 hpi) (D and D′) pre-neoplastic cells is shown in green. (C) HRASG12V-GFP-expressing superficial clones (basal cells shown in magenta) in 3 dpf larva (asterisk, C″) and scanning electron microscopy (72 hpi) (C″′). (D) HRASG12V-GFP-expressing basal clones in 5 dpf larva (superficial cells in magenta) (asterisk in D″ and scanning electron microscopy in D″′). (E–H) Mosaic expression of oncogenic HRASG12V in skin cells compared with control GAP-43 GFP expression results in recruitment of neutrophils (magenta; E) and macrophages (red; F) 48 hpi, quantified in (G) and (H), respectively. See also Figure S1. (I) Knockdown of both neutrophils and macrophages (with PU.1 and granulocyte colony stimulating factor [GCSF] MOs) inhibits superficial pre-neoplastic cells growth (GFP) in 48 hpi larvae. Scale bars: 100 μm (A′, A″, B′, B″, C′, C″, D′, D″, E, F, and I); 20 μm (A″′, B″′, C″′, and D″′). Graphs display mean ± SEM.

In contrast with adult mammalian skin, larval zebrafish skin consists of only two epithelial cell layers, the outer superficial epidermal cell layer and the inner basal epidermal cell layer, the latter tethered to the basement membrane (largely consisting of collagen IV and laminin) (Hynes, 2012) by hemi-desmosomal junctions (Fischer et al., 2014, Le Guellec et al., 2004). Directly beneath the basement membrane there is a further layer of ECM largely consisting of collagen I (depicted in Figure 1A). To analyze normal healthy skin architecture in larvae, we crossed lines expressing cytoplasmic GFP in superficial epidermal cells (Gong et al., 2002, Imboden et al., 1997) with those expressing tdTomatoCAAX in basal cells (Lee et al., 2014, Morris et al., 2018) (Figures 1A, 1A′, and 1A′′). Scanning electron microscopy reveals the polygonal, pavement-like pattern of superficial cells, with orifices where goblet cells, mucous-secreting cells of wet epithelium, are visible at their interfaces (Figure 1A′″).

To study events during cancer initiation in skin, we used three models to express HRAS^G12V^ under different promoters: the *kita* promoter drives expression in melanocytes and goblet cells (Figure 1B) (Santoriello et al., 2010) (model referred to as kita:RAS), the *keratin4* promoter drives expression in superficial cells (K4:RAS) (Ramezani et al., 2015) (Figure 1C), and the *krtt1c19e* promoter drives expression in basal cells (K19:RAS) (Figure 1D). All three models make use of the gal4-UAS system, and two are 4-hydroxytamoxifen (4OHT) inducible for temporal control of mosaic HRAS^G12V^-GFP expression (Ramezani et al., 2015). We observe how clones of each of these HRAS^G12V^-GFP-expressing lineages disrupt normal skin architecture: kita:RAS leads to proliferation of goblet cells (Figure 1B) sitting within the *tdTomato*-expressing basal cell layer (Figure 1B′′). Scanning electron microscopy shows how these proliferating goblet cells disturb the otherwise continuous superficial epidermal layer (Figure 1B′″). By comparison, mosaic expression of K4:RAS (Figures 1C′ and 1C′′) results in superficial cell clones that are more disruptive, leading to a general mixing of epithelial cells between their two originating layers (Figure 1C′′). Scanning electron microscopy images show considerable disorganization and protruding cells (Figure 1C′″). Similarly, pronounced disorganization of the skin is apparent in the K19:RAS model (Figures 1D′ and D′′), where both basal cells and superficial cells protrude, confirmed by scanning electron microscopy (Figure 1D′″).

### Pre-neoplastic Skin Cells Recruit High Numbers of Innate Immune Cells That Are Essential for Their Growth

**Figure 2:**
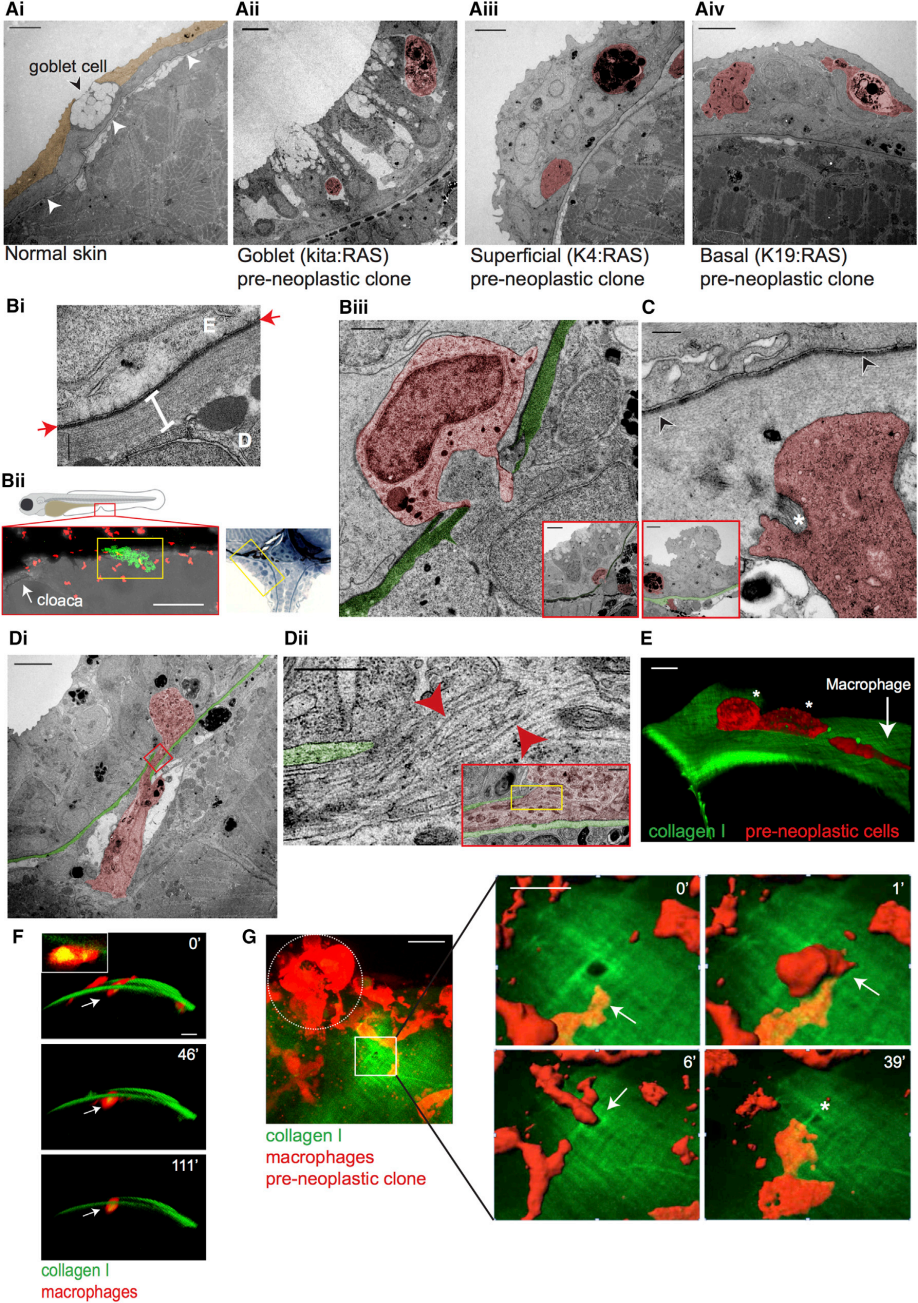
Correlative Light and Electron Microscopy of Immune Cells Entering the Epidermis (Ai) Transmission electron microscopy (TEM) of 5 dpf WT larval skin; superficial epidermal layer (sepia), basal layer beneath, and the basement membrane, visible as a thin dark line (arrowheads) and goblet cell. (ii) TEM of kita:RAS clone (16 dpf), and superficial (iii) and basal (iv) clones in 5 dpf (72 hpi) larvae, shows immune cells (false colored red) within the clones. (Bi) TEM of larval BMZ. Red arrows indicate the basement membrane; the white bar indicates collagen I, E is the epidermis above, and D is the dermis below the BMZ. (ii and iii) CLEM; confocal image (ii, lower left) and methylene blue-stained section (ii, right) show where clone and immune cell interactions take place (yellow boxes) near the cloaca (arrow in ii). (iii) Granulocyte (red) in a breach in the BMZ (green) beneath a goblet cell clone (see inset) in 10 dpf larva. (C) Neutrophil (red) protrusions surrounding bundled collagen I (asterisk) below the BM (arrowheads) beneath a pre-neoplastic superficial cell clone (in inset BM zone is colored in green) in 5 dpf (72 hpi) larva. (Di) A macrophage (red), containing collagen, spans a breach in the BM (green) beneath a basal cell clone in 5 dpf (72 hpi) larva. (ii) High-magnification view corresponding to yellow box in red inset, which, in turn, corresponds to red box in (i). Red arrowheads indicate collagen fibrils. (E) Confocal imaging of a 5 dpf/48 hpi larva shows a macrophage (red, see white arrow) above the collagen I layer (green) approaching two pre-neoplastic basal cells (red, see white asterisks). (F) Confocal imaging of a macrophage (white arrows) containing engulfed collagen (yellow) in the collagen I-GFP layer in 5 dpf (48 hpi) larva. Inset: a single z stack shows collagen I-GFP (yellow) within the macrophage. (G) Stills from a time-lapse video (minutes in top right corner) show a macrophage (red) squeezing through a pre-existing hole in the collagen I layer (green) beside a superficial pre-neoplastic clone on the left (red, dotted white circle) in 5 dpf (72 hpi) larva. See also Video S1. Scale bars: 5 μm (Ai–Aiv, Biii and C [insets], Di, and G [video stills]); 500 nm (C); 100 nm (Dii); 1 μm (Dii [inset] and Biii); 10 μm (E and F); 20 μm (G); 500 nm (Bi); and 100 μm (Bii).

Pre-neoplastic kita:RAS cells in larval zebrafish skin lead to an inflammatory response (Feng et al., 2010, Freisinger and Huttenlocher, 2014). We found an increased recruitment of both neutrophils and macrophages to HRAS^G12V^-expressing clones in both superficial and basal cell models also by 48 h postinduction (48 hpi) (Figures 1E and 1F; Figure S1), quantified in Figures 1G and 1H (superficial) and Figure S1 (basal). To investigate the significance of inflammatory cell recruitment, we performed morpholino (MO)-mediated knockdown of neutrophils and macrophages (Feng et al., 2012). A combination of pu.1 (Rhodes et al., 2005) and gcsfr1 (Liongue et al., 2009) MOs results in a significant decrease in pre-neoplastic cell growth (Figure 1I). Examination of transmission electron microscopy (TEM) sections from each cancer model revealed morphologically distinct innate immune cells within or in the vicinity of pre-neoplastic clones (Figures 2Aii–2Aiv), whereas away from pre-neoplastic cells, immune cells in the epidermal layer were very rare (Figure 2Ai), supporting our light microscopy imaging and quantification (Figures 1E–1H; Figure S1).

### Capturing Immune Cells as They Traverse the Basement Membrane Zone to Access Pre-neoplastic Skin Cells

Both light and TEM data described above indicate that innate immune cells make direct contact with pre-neoplastic cells in the larval skin. However, it is unclear how they gain access to these cells because they are separated by the BMZ matrix barrier (Figure 2Bi). By fixing larvae when our live-imaging studies indicate that immune cells have arrived at a clone of pre-neoplastic cells, we can perform CLEM to capture instances where immune cells have just breached the BMZ directly beneath HRAS^G12V^-expressing clones (Figures 2Bii and 2Biii). We also observe immune cells with bundled collagen between cell protrusions suggesting collagen degradation at the BMZ (Figure 2C). And we also show immune cells spanning a breach through the BMZ beneath a pre-neoplastic basal cell clone (Figure 2Di). TEM indicates examples of encapsulated collagen fibrils within breaching macrophages (Figure 2Dii). The epidermal-derived interstitial collagen I of the BMZ can be distinguished by confocal imaging of a collagen I-GFP-transgenic fish (Morris et al., 2018), where collagen I-GFP is expressed under the control of the basal epithelial cell-specific promoter K19. We crossed this collagen I-GFP-transgenic fish with one expressing mCherry in macrophages and mosaically induce HRAS^G12V^ in basal (Figure 2E) or superficial (Figure 2G) cells. Lateral view images from a video of such larvae reveal macrophages containing collagen I-GFP as they move through the collagen I layer (Figure 2F, inset). Macrophages “sit” for periods of up to 120 min within the matrix layer (Figure 2F).

Interestingly, confocal imaging of the collagen I layer in the vicinity of pre-neoplastic superficial clones also reveals occasional small pre-existing holes near the clone (Figure 2G). We captured macrophages traversing through such pre-existing holes (Figure 2G; Video S1) as they gain access to the epithelial layer; strikingly, these traverses are rapid, taking between 5 and 30 min, which is faster than the time required for active degradation of the matrix (Sabeh et al., 2009). These pre-existing holes were presumably generated previously, either proteolytically or mechanically, and subsequently used by immune cells as a route through the basement membrane to reach the epidermis. A similar “tunneller and follower cell” scenario is described for cancer-associated fibroblasts (CAFs) as they proteolytically degrade matrix providing a route for cancer cells to metastasize (Gaggioli et al., 2007), and also may enable xenografted cancer cell migrations as neutrophils first deform collagen matrix in the vicinity of larval zebrafish cancer explants (He et al., 2012). A recent *in vitro* study describes immune cells sampling their vicinity for large pores in the matrix, allowing them to choose paths of least resistance (Renkawitz et al., 2019). The rapidly traversed holes we observe occasionally remain open but sometimes shrink in size after the immune cell has passed through (Figure 2G). The speed of traversing may explain why we so rarely capture these short windows of opportunistic migratory activity.

Video S1. Capturing the Minutes as a Macrophage Opportunistically Squeezes through an Already Established Hole in the Collagen I Matrix (Green) Layer of the BMZ, Related to Figure 2G

**Figure 3:**
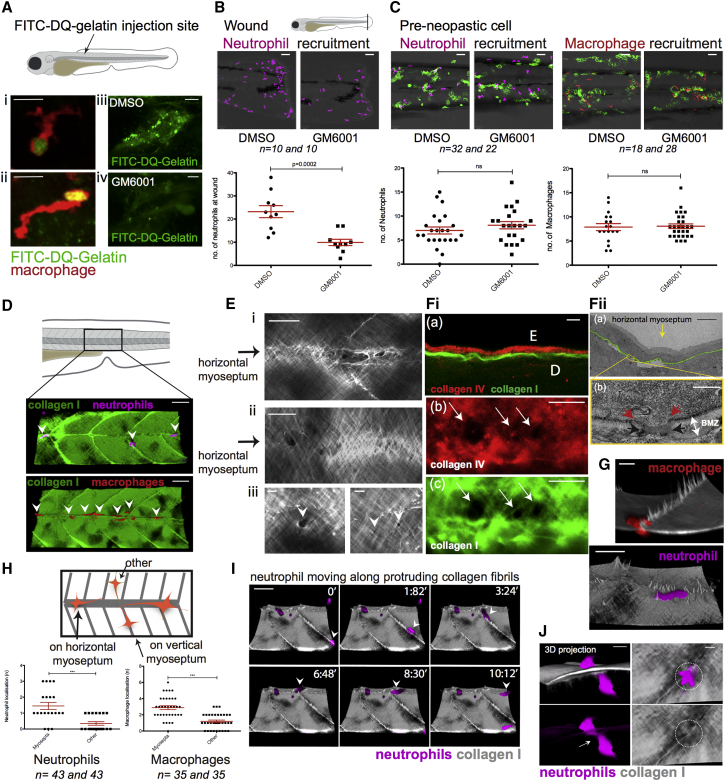
Weak Spots in the BM Barrier Layer Allow Opportunistic Crossing of Immune Cells into the Epidermis (A) De-quenched fluorescein isothiocyanate (FITC)-gelatin in 3 dpf larva indicates MMP activity (green or yellow) at the leading edge of macrophages (red; i and ii). GM6001 inhibits MMP activity in whole somite (iv versus iii). (B) GM6001 inhibits neutrophil recruitment to tail fin wound, but does not inhibit neutrophil (magenta) or macrophage (red) recruitment to pre-neoplastic cells in 3 dpf (24 hpi) larvae (C). See also Figures S2A and S2B. (D) Neutrophils and macrophages preferentially move along the horizontal myoseptum (indicated with arrowheads) in wild-type 5 dpf larval skin. See also Figures S2C and S2D. (E) Collagen along the horizontal myoseptum of 5 dpf larva shows altered structure and gaps or weak spots (i and ii). Higher-magnification view illustrates variation in size of gaps along the horizontal myoseptum (iii, white arrowheads). See also Figure S2E. (Fi) Immunostaining of collagen I (green) and collagen IV (red) at the epidermal (E) dermal (D) interface (a) reveals concomitant holes in collagen IV (b) and collagen I (c) along the horizontal myoseptum of 5 dpf larvae. (ii) TEM of 5 dpf WT larval skin shows a gap through the BMZ at the horizontal myoseptum (yellow arrow in a). Red arrowheads indicate the margins of the BM gap; black arrows define margins of disrupted collagen I in the same location (b). (G) Macrophages (red, 14 dpf) and neutrophils (magenta, 5 dpf) crawling adjacent to collagen I fiber “tracks” (gray). (H) Schematic and quantification of neutrophils and macrophages within the flank of 3 dpf larvae. (I) Still series from a video shows neutrophil (magenta) migrating along the protruding collagen I fibers (gray) at the myosepta (arrowhead) in 5 dpf larva. See also Video S2. (J) Neutrophil (magenta) squeezes (arrow) through collagen I at myoseptum in a larva 14 dpf. Scale bars: 10 μm (Ai, Aii, Fib, and Fic); 20 μm (Aiii, Aiv, Ei, Eii, G, and J); 5 μm (Eiii, Fia, and Fiia); 50 μm (B, C, D, and I); 400 nm (Fiib). Graphs display mean ± SEM.

To investigate the importance of proteolytic degradation of the BMZ by immune cells to access epidermal pre-neoplastic clones, *in vivo* “zymography” studies visualized local matrix metalloproteinase (MMP) activity (Travnickova et al., 2015). Highly de-quenched (DQ) fluorescein-labeled gelatin was injected into the flank of 3 days postfertilization (dpf) larvae, and fluorescence resulting from degradation of the gelatin was observed at the leading edges of macrophages, suggesting MMP activity by these cells (Figure 3Ai and 3Aii) that can be blocked by MMP inhibitor GM6001 (Figure 3Aiii and 3Aiv). Treatment of larvae with GM6001 inhibits neutrophil migration to tail fin wounds as described previously (Hall et al., 2014) (Figure 3B); however, the same treatment did not inhibit immune cell recruitment to pre-neoplastic cells (Figure 3C). Similar is true for larvae treated with a pan-protease inhibitor cocktail or a neutrophil elastase inhibitor (Sivelestat) (Figures S2A and S2B). These data suggest that although immune cells may be able to proteolytically burrow through the matrix, they can also traverse in ways that are independent of proteolysis. Indeed, T cells move in an amoeboid fashion through a 3D matrigel substrate, pushing pseudopodial extensions through pre-existing collagen gaps, if proteolysis is blocked (Wolf et al., 2003). Similarly, in a 3D *in vitro* model of carcinoma, CAFs were shown to remodel and soften the matrix between themselves and human colon cancer cells enabling cancer cell invasion, also in a protease-independent fashion (Glentis et al., 2017).

### Opportunistic Access to the Epidermis by Immune Cells Is through Pre-existing “Weak Spots” along the Horizontal Myoseptum and Leads to Bigger Clones of Pre-neoplastic Clones Locally

In order to better understand how immune cells traverse the BMZ in a protease-independent manner, we investigated how the few neutrophils and macrophages in wild-type (WT) larval skin gain access to the epidermis. Live imaging of otherwise WT, collagen I-GFP larvae with fluorescently labeled neutrophils and macrophages reveals protruding collagen I fibers along the horizontal and vertical myosepta, which may provide a preferred route for immune cell migration (Figure 3D; Figures S2C and S2D; and quantified in Figure 3H). High-resolution imaging of fibrils shows disruptions in collagen organization, leaving weak spots in the collagen I layer; on average we see two “holes” per somite, ranging from 1 to 4 μm in diameter (Figure 3E) in all larvae examined and in older fish also along the transverse myosepta (Figure S2E). Co-immunostaining of collagen I-GFP and endogenous collagen IV (the main component of the lamina densa of the basement membrane) shows a co-incidence of holes (of 17 collagen I holes analyzed, 15 show a clear concomitant collagen IV disruption), supporting the use of the transgenic (Tg) collagen I-GFP fish as a tool to live-image the BMZ and as a proxy for indicating breaches through the matrix barrier layers (Figure 3Fi). TEM studies also show co-incidental disruption of the BMZ collagen I matrix and the BM itself (Figure 3Fii). We observe collagen I fibers protruding down into the tissue along the myosepta, partitioning the developing myotomes, and possibly confining innate immune cells to “highways” leading them along regions where the BMZ has weak spots (Figures 3G, 3I, and 3J; Video S2; Figure S2D).

Video S2. Neutrophils (Purple) Moving along the Underside of Collagen I Matrix (Gray) Layer of the BMZ, Apparently Guided by Myoseptal Fibrils Tracks, Related to Figure 3I

**Figure 4:**
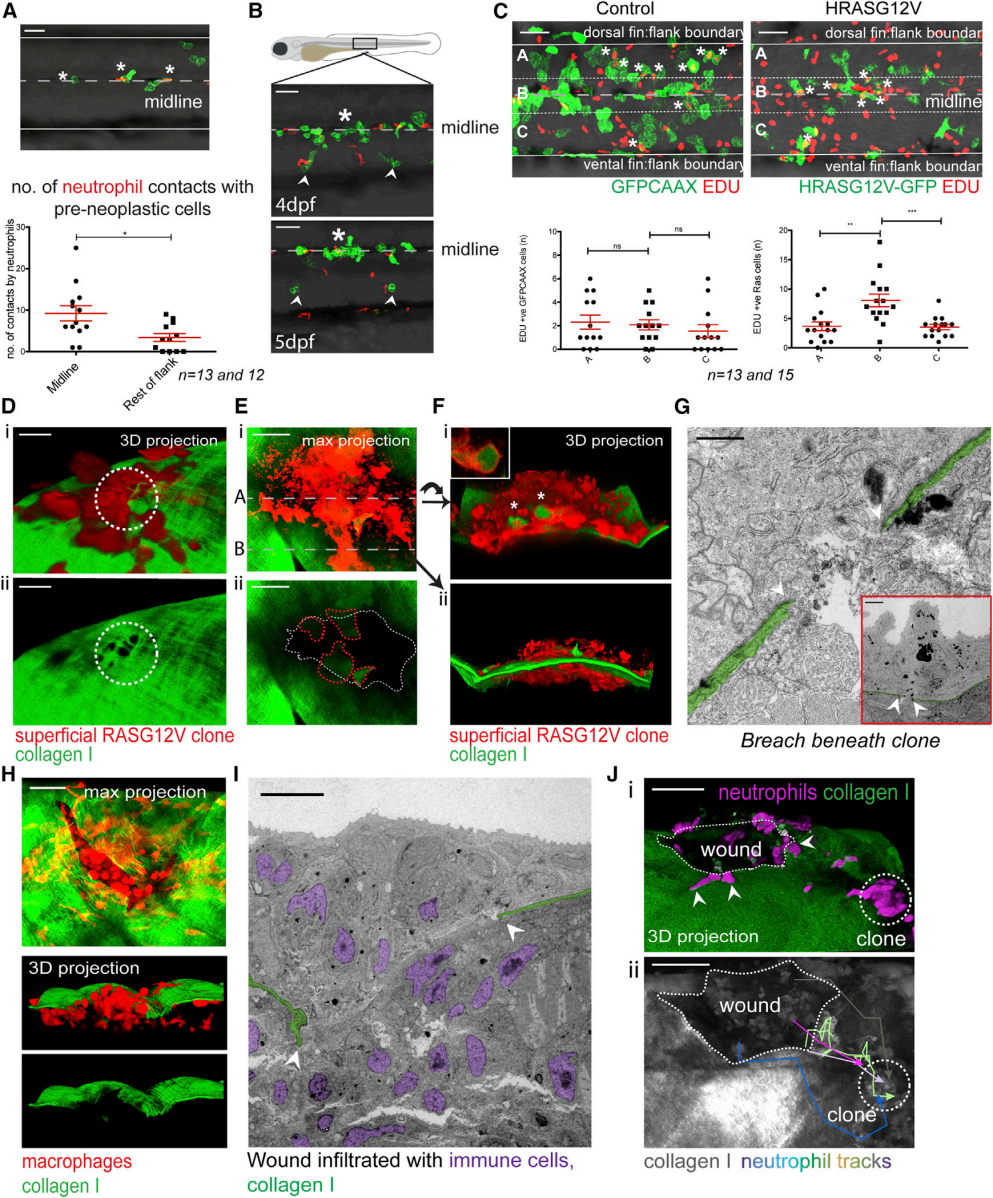
Immune Cells Access Epidermal Clones through Portals in the Basement Membrane (A) Neutrophil and pre-neoplastic cell contacts (asterisks) along the horizontal myoseptum compared with elsewhere in the flank 8 hpi, over a 3-h period. (B) Proliferation of clones (asterisk) along the horizontal myoseptum compared with clones farther away (arrowheads). (C) Example of EDU staining of control, GFPCAAX-expressing basal cells (left) versus GFP-expressing HRASG12V basal cells (right) at 18 hpi. Quantification of double EDU (red) and GFP +ve cells in indicated zones: A, B, and C. (D–G) Degradation of the BMZ beneath later stage pre-neoplastic cell clones. (Di) A superficial HRASG12V-expressing clone (red) on collagen I-GFP with holes in the collagen I layer immediately beneath the clone (white dotted circle, Dii) in a larva 6 dpf (96 hpi). (E) Degraded collagen I-GFP zone beneath a large HRASG12V-expressing superficial clone (red) in a larva 5 dpf (96 hpi; white dotted line in Eii). Lumps of collagen I within the pre-neoplastic cells are outlined (red dotted line). (Fi) A transverse 3D view of the clone along dotted line A in (Ei). GFP-collagen I within pre-neoplastic cells (asterisks and inset in Fi). See also Figures S3A and S3B. 3D view of clone along dotted line B in (Ei) shows invasion of the clone through the collagen I-GFP layer in (Fii). (G) TEM shows BMZ degradation (white arrowheads) beneath a large HRASG12V-expressing superficial cell clone (120 hpi) in 6 dpf larvae. See also Figure S3C. (H) Maximal projection confocal image of a flank wound in a larva 7 dpf, 2 days postinjury, shows a defect in the collagen I-GFP layer and recruited macrophages (red). 3D projection shows macrophages (red) below and above the matrix breach. (I) CLEM shows breach (arrowheads) in the BMZ (green) with invading immune cells (purple nuclei). (Ji) Neutrophils (magenta) escaping the wound (arrowheads) and crawling over collagen I layer toward pre-neoplastic superficial clone (dotted circle) as indicated by neutrophil tracks in (Jii). See also Figure S4. Scale bars: 50 μm (A–C, H, and J); 10 μm (D and I); 30 μm (E); 1 μm (G), 5 μm (G inset). Graphs display mean ± SEM.

If these ready-made holes provide favored sites where immune cells can access the epidermis, one might expect that clones of pre-neoplastic cells lying in close proximity to the horizontal myoseptum would be at a competitive advantage for immune cell visits, and consequently for the trophic signals that these cells deliver. Indeed, there are significantly more neutrophil contacts with clones at the midline compared with clones located in adjacent regions (Figure 4A), and clones grow faster along the midline (Figure 4B). To quantify this, we compared the proliferation of pre-neoplastic cells in clones near to the midline versus more distant clones by performing 5-ethynyl-2′-deoxyuridine (EDU) staining of HRAS^G12V^-expressing basal skin cells. In control larvae, the number of proliferating cells is equally distributed across the flank, but in HRAS^G12V^-expressing larvae, proliferation is increased in clones along the midline of the fish. This supports the concept that pre-neoplastic clones in close proximity to the horizontal myoseptum receive more immune cell visits driving increased proliferation (Figure 4C).

### Collagen Uptake by Larger Pre-neoplastic Clones or Mechanical Damage to the Skin Generates Further, More Extensive BMZ Breaches

The regions of the BMZ beneath growing clones of pre-neoplastic cells are of considerable interest because these are the regions where the BM becomes eroded on tumor invasion. In the larval cancer models, as pre-neoplastic clone diameter increases to 30–50 μm, individual holes beneath them begin to coalesce (Figure 4D). As clone diameters increase to greater than 100 μm, large patches with missing collagen extend beneath them (Figure 4E). Associated with this matrix loss, collagen I-GFP (which is expressed only by basal epithelial cells) is observed, not only within immune cells, but also within the superficial pre-neoplastic cells (Figure 4F, inset; Figures S3A and S3B), suggesting active engulfment of matrix, which might affect cancer cell behavior (Egeblad et al., 2010), but which also provides a further potential route for epidermal access by immune cells. TEM of larvae with bigger pre-neoplastic clones confirms this missing or disrupted BM (Figure 4G; Figure S3C). Unsurprisingly, these larger clones, where areas of the BM are missing, are most frequently located along the horizontal myoseptum (80% of these clones in 30 fish lie on the horizontal myoseptum) (Figures 4E and 4G; Figure S3C), suggesting that at sites where there is a pre-existing altered or weakened matrix barrier, there is an increased likelihood of subsequent cancer invasion.

Importantly, damage to the epidermis, for example, resulting from diagnostic needle biopsy of patients or surgery, will generate matrix breaches of considerable size and duration. We have previously shown that tissue damage impacts on the inflammatory response to clones of pre-neoplastic cells in the vicinity of a wound through release of inflammatory cell attractants (Antonio et al., 2015), and there is considerable literature on how wounding may exacerbate cancer progression (Krall et al., 2018, Schäfer and Werner, 2008, Szalayova et al., 2016). By wounding collagen I-GFP fish, we observe how such a lesion results in a significant breach in the matrix barrier layer, and thus provides another large routeway for immune cells to access the epidermis (Figure 4H; Figure S4). CLEM of such wounds reveals invading immune cells accessing the epidermis at the wound margin (Figures 4H and 4I). Live-imaging studies of wounded larvae suggest that although both neutrophils and macrophages can access and enter the epidermis at these ECM barrier breaches, largely only neutrophils progress onward to migrate beyond the wound margin toward pre-neoplastic cells (Figure 4J) (Antonio et al., 2015). We propose that wound-mediated recruitment of immune cells and their subsequent impact on nearby pre-neoplastic cells is not only a consequence of damage attractants, but also because tissue damage provides a large portal through the ECM barrier for immune cells to gain direct access to cancer cells.

Our observations indicate that there may be multiple ways for neutrophils and macrophages to traverse the basement membrane barrier to access pre-neoplastic cells in the epidermis. Proteolytic degradation of matrix is not essential because there are pre-existing, naturally occurring weak spots in the ECM barrier that act as opportunistic portals for immune cells to move from connective tissue into the epidermis; in healthy skin these portals are used for immune surveillance (Figure S4). These weak spots in the BMZ beneath the larval epidermis share similarities with the pattern of matrix distribution around postcapillary venules, where low expression regions are the preferential sites for immune cell extravasation or diapedesis through the vessel wall (Voisin et al., 2010). In embryonic tissues, breakdown of the BM enables, and even directs, important cell migrations, as, for example, anchor cell migration leading to fusion with vulval cells in *C. elegans* (Sherwood and Sternberg, 2003), and these developmental invasions may share mechanisms with cancer cell invasion. Previous studies highlight the usefulness of zebrafish as a model to study human BM diseases (Feitosa et al., 2011, Li et al., 2011) and have characterized and compared BM components between mammals and zebrafish (Nauroy et al., 2018, Nauroy et al., 2019). Our observations in larval tissues will need verification in adult mammalian tissues in order to be of clinical relevance, but, for example, microperforations in the basement membrane of the bronchial airway and small intestine have previously been described (Howat et al., 2001, Takeuchi and Gonda, 2004), and in pathological conditions such as inflammatory bowel disease these may become the precursors of portals for immune cell influx into tissues that, in turn, often precede malignancy (McAlindon et al., 1998, Spenlé et al., 2012).

Our data show that immune cells can take advantage of the easiest routes through the barrier ECM to access the epithelium in which pre-cancer cells reside. These portals may be small, in otherwise undamaged BM, or larger gaps that are generated as a consequence of cancer erosion or biopsy or surgical wounding, and we show that this access of immune cells to cancer cells is rate limiting for cancer progression (Figure S4). Further studies of these various portals will highlight their usefulness as potential biomarkers for likely cancer progression and as therapeutic targets for cancer prevention.

## STAR★Methods

### Key Resources Table

**Table:** 

REAGENT or RESOURCE	SOURCE	IDENTIFIER
**Antibodies**		

Rabbit monoclonal anti-GFP	Cell Signaling Technology	Cat#2956
Alexa Fluor 488 Goat anti-Rabbit	Invitrogen	Cat#A-11008; RRID: AB_143165
Rb Ab to collagen IV	AbCam	Cat# Ab6586
Mouse monoclonal anti-GFP	Abcam	Cat# Ab1218
Alexa Fluor 546 goat anti-rabbit	Invitrogen	Cat# A11035; RRID: AB_2534093
Alexa Fluor 488 goat anti-mouse	Invitrogen	Cat# A11029; RRID: AB_2534088

**Chemicals, Peptides, and Recombinant Proteins**		

4-hydroxytamoxifen	Sigma-Aldrich	Cat#T176
GM6001	Millipore	Cat#CC1010
FITC-gelatin	AnaSpec	Cat#AS-85145
Sivelestat sodium salt	Tocris	Cat#3535
Leupeptin	Tocris	Cat#1167
Pepstatin A	Tocris	Cat#1190
Aprotinin	Tocris	Cat#4139
E-64c	Caymen Chemical	Cat#10007964

**Critical Commercial Assays**		

Click-iT Plus EdU Alexa Fluor 647 Imaging Kit	Life Technologies	Cat#C10640

**Experimental Models: Organisms/Strains**		

*Danio rerio:* Tg(*krt8:GFP)gz7*	Gong et al., 2002	ZFIN ID: ZDB-ALT-080207-1
*Danio rerio:* Tg*(krt19:tdTomatoCAAX)*	Lee et al., 2014, Morris et al., 2018	ZFIN ID: ZDB-ALT-140424-2
*Danio rerio:* Tg(*lyz:DsRed*)*nz50*	Hall et al., 2007	ZFIN ID: ZDB-ALT-071109-3
*Danio rerio:* Tg*(mpeg1:mCherry)gl23*	Ellett et al., 2011	ZFIN ID: ZDB-ALT-120117-2
*Danio rerio:* Tg(*UAS:GAP43-GFP*)*u300*	Kajita et al., 2010	ZFIN ID: ZDB-ALT-101118-1
*Danio rerio:* Tg(*UAS:eGFP*)	Santoriello et al., 2010	N/A
*Danio rerio:* Tg*(6xUAS:mCherry-HRAS*^*G12V*^*)*	This manuscript	N/A
*Danio rerio:* Tg*(5XUAS:eGFP-HRAS*^*V12*^*)io6*	Santoriello et al., 2010	ZFIN ID: ZDB-ALT-090702-2
*Danio rerio:* Et(*kita:GalTA4, UAS:mcherry*)*hzm1*	Distel et al., 2009	ZFIN ID: ZDB-ALT-090702-3
*Danio rerio:* Tg*(kita:Gal4;UAS:HRAS*^*G12V*^*-GFP)*	Santoriello et al., 2010, Feng et al., 2010	N/A
*Danio rerio:* Tg(*krt19:col1α2-GFP*)	Morris et al., 2018	N/A
*Danio rerio:* Tg*(krt19:col1α2-GFP;lyz:dsRed)*	This manuscript	N/A
*Danio rerio:* Tg(*krt19:col1α2-GFP*;*mpeg1:mcherry)*	This manuscript	N/A
*Danio rerio:* Tg*(krt19:col1α2-GFP;UAS:mCherry-HRAS*^*G12V*^*)*	This manuscript	N/A
*Danio rerio:* Tg*(krt19:col1α2-GFP;mpeg1:mCherry; UAS:mCherry-HRAS*^*G12V*^*)*	This manuscript	N/A
*Danio rerio:* Tg*(krt19:col1α2-GFP; lyz:dsRed;UAS:mCherry-HRAS*^*G12V*^*)*	This manuscript	N/A

**Oligonucleotides**		

*pu.1* 5′- GATATACTGATACTCC ATTGGTGGT-3′	GeneTools LLC	Rhodes et al., 2005
*gcsfr* 5′-AATGTTT CGCTTACTTTGAAAATGG-3′	GeneTools LLC	Liongue et al., 2009

**Recombinant DNA**		

6xUAS 5E	Dr. Dirk Sieger, Edinburgh	N/A
mCherry-HRAS^G12V^ ME vector	This manuscript	N/A
polyA 3E vector	zebrafish Tol2kit, Kwan et al., 2007	N/A
pDestTol2CG vector	zebrafish Tol2kit, Kwan et al., 2007	N/A
*pTol2-krt4:KalTA4-ERT2;cmlc2:eGFP*	Ramezani et al., 2015	N/A
*pTol2-krt19:KalTA4-ERT2;cmlc2:eGFP*	This manuscript	N/A

**Software and Algorithms**		

Volocity	PerkinElmer	http://www.perkinelmer.co.uk/lab-products-and-services/resources/whats-new-volocity-6-3.html
ImageJ / Fiji	Fiji	http://fiji.sc/
Imaris	Bitplane (Oxford Instruments)	https://imaris.oxinst.com
Photoshop	Adobe	http://www.adobe.com/uk/products/photoshop.html
Illustrator	Adobe	http://www.adobe.com/uk/products/illustrator.html
Prism	GraphPad	https://www.graphpad.com/scientific-software/prism/

**Other**		

Glass bottomed Matek microscopy dish, 35mm	MatTek Corp	Cat#S319281
Leica SP8 AOBS confocal laser scanning microscope attached to a Leica DM6000 upright epifluorescence microscope	Leica	https://www.leica-microsystems.com/products/confocal-microscopes/p/leica-tcs-sp8/
Leica TCS SP8 AOBS confocal laser scanning microscope attached to a Leica DMi8 inverted epifluorescence microscope	Leica	https://www.leica-microsystems.com/products/confocal-microscopes/p/leica-tcs-sp8/
Transmission electron microscope FEI Tecnai 12-FEI 120kV BioTwin Spirit	Tecnai (Thermo Fisher Scientific)	https://www.fei.com/tecnai-upgrades/
Scanning electron microscope FEI Quanta 200FEG SEM	FEI (Thermo Fisher Scientific)	https://www.fei.com/products/sem/quanta-sem/ (discontinued)
Critical point dryer, Leica EM CPD300	Leica Microsystems	https://www.leica-microsystems.com/products/sample-preparation-for-electron-microscopy/p/leica-em-cpd300/
Sputter coater, Emitech K575X	Emitech (Quorum tecnologies)	https://www.quorumtech.com/previous-products/coaters-and-evaporators (discontinued)

### Contact for Reagent and Resource Sharing

Further information and requests for resource and reagents should be directed and will be fulfilled by the Lead Contact, Paul Martin (Paul.Martin@bristol.ac.uk).

### Experimental Model and Subject Details

#### Zebrafish husbandry

Adult zebrafish (*Danio rerio*) were maintained as previously described (Westerfield, 2007). All experiments were conducted with local ethical approval from the University of Bristol and in accordance with UK Home Office regulations (Guidance on the Operations of Animals, Scientific Procedures Act, 1986). All zebrafish lines are listed in Table S1. Our collagen lines were crossed onto a Casper background to prevent auto-fluorescence from melanocytes during confocal imaging. To induce mosaic HRAS^G12V^ expression in the collagen lines, Tg*(krt19:col1α2-GFP;mpeg:mCherry)* fish were crossed with Tg*(6xUAS:mCherry-HRAS*^*G12V*^*)* fish to make the final Tg*(krt19:col1α2-GFP;mpeg:mCherry;UAS:mCherry-HRAS*^*G12V*^*)* transgenic line. These fish we then outcrossed with Tg*(krt19:col1α2-GFP, mpeg:mCherry)* to generate homozygous collagen I-GFP-expressing larvae which we subsequently microinjected with *krt4:KalTA4-ER*^*t2*^ or *krt19:KalTA4-ER*^*t2*^ and treated with 4-hydroxytamoxifen (4OHT), as described below, to induce mosaic HRAS^G12V^-expression in either superficial or basal cells respectively.

### Method Details

#### Microinjection

To generate krt4-superficial or krt19-basal mosaic cancer lines, 12.5 to 25ng of *pTol2-krt4:KalTA4-ERT2;cmlc2:eGFP* or *pTol2-krt19:KalTA4-ERT2;cmlc2:eGFP* was injected together with 50ng/μl of purified capped Tol2 mRNA into 1 cell-stage Tg(*UAS:RAS*^*G12V*^*-GFP*) embryos as described previously (Ramezani et al., 2015). Injected larvae were subsequently treated with 5 μM 4OHT (Sigma-Aldrich, T176) to induce mosaic HRAS^G12V^ expression in either superficial or basal cells. The length of time of expression of HRAS^G12V^ can be controlled and is described in terms of hours post induction (hpi).

#### Constructs

*pTol2-UAS:RAS*^*G12V*^*-mCherry;cry:CFP* was made using the modular MultiSite Gateway cloning strategy, cloning the 6xUAS 5E vector (kind gift from Dr. Dirk Sieger, Edinburgh), mcherry-HRAS^G12V^ ME vector and polyA 3E vector into pDestTol2CG vector from the zebrafish Tol2kit (Kwan et al., 2007) that contains a *cry:eCFP-pA* to enable F0 screening. *pTol2-krt19:KalTA4-ERT2;cmlc2:eGFP* was made as described previously for *pTol2-krt4:KalTA4-ERT2;cmlc2:eGFP* (Ramezani et al., 2015), using the krt19 promoter.

#### Generation of Tg(6xUAS:mCherry-HRASG12V) larvae

12.5 to 25ng of *pTol2-UAS:HRASG12V-mCherry;cry:CFP* construct together with 50ng/μl purified capped Tol2mRNA was injected into one cell stage Casper embryos. Injected larvae were screened for CFP positive eyes at 3-5dpf by fluorescent microscopy and F1 fish were screened for germline transmission. Positively identified founder fish were grown to adulthood and F2 generations were crossed to Et(*kita:GalTA4,UAS:mCherry*) fish to check for mCherry-expressing HRASG12V goblet cell clones. Additionally, these fish were used for microinjection of *krt4:KalTA4-ER*^*t2*^ or *krt19:KalTA4-ER*^*t2*^ to mosaically induce mCherry-expressing superficial or basal cell clones, and crossed onto Tg(*krt19:col1α2-GFP;mpeg:mCherry*) fish for live imaging of macrophage movement in relation to collagen I in response to pre-neoplastic skin cell growth.

#### Morpholino experiments

All morpholinos were obtained from GeneTools LLC. Morpholinos were suspended in distilled water to a concentration of 1mM. 0.5nl drops of 0.25 μM *pu.1* + *gcsfr* MO were injected into one-cell stage Tg*(UAS:RAS*^*G12V*^*-GFP)* embryos together with 25 ng of *krt4:KalTA4-ER*^*t2*^, to knockdown both neutrophils and macrophages and to simultaneously induce mosaic HRAS^G12V^ expression in superficial cells.

The following morpholinos were used: *pu.1* 5′- GATATACTGATACTCC ATTGGTGGT-3′ (Rhodes et al., 2005) and *gcsfr* 5′-AATGTTT CGCTTACTTTGAAAATGG-3′ (Liongue et al., 2009).

#### Wounding

2dpf tg(*UAS:RAS*^*G12V*^*-GFP;lyz:dsRed)* larvae were treated with 10 μM GM6001 or DMSO for 48 hr. At 4dpf a tail fin wound was made with a sterile scalpel at the level of the posterior edge of the notochord. Fish were imaged between 5-6hrs post wounding and analyzed for neutrophil recruitment. Experiments were performed blinded (Figure 3B).

Four days post fertilization larvae were wounded with a 30G hypodermic needle on their flank either directly above the cloaca or, if there was a pre-neoplastic clone nearby, further away from the clone to prevent wounding of the clone itself. Recruitment of neutrophils was imaged 1 day post wounding (dpw) and recruitment of macrophages at 2dpw (Figures 4H–4J).

#### Drug treatments

*krt4:KalTA4-ER*^*t2*^ or *krt19:KalTA4-ER*^*t2*^ microinjected larvae were treated with 5 μM 4-hydroxytamoxifen (Sigma-Aldrich, T176) at either 1dpf or 2dpf between 24 and 120hours depending on the experiment. For recruitment of neutrophils and macrophages to pre-neoplastic cells, larvae were treated for 48hrs at 1dpf (Figures 1E–1I; Figure S1). To initiate larger pre-neoplastic clones, larvae were treated from 2dpf between 72 and 120hrs (Figures 2G and 4D–4G). For quantifications of neutrophil contacts, larvae were treated for 8hrs and for EDU experiments, larvae were treated for 18hrs.

10 μM GM6001 (Millipore, CC1010) was either co-injected in the flank of 3dpf larvae with FITC-gelatin (AnaSpec, AS-85145) (Figure 3A) or 2dpf larvae were pre-treated with 10 μM GM6001 or DMSO by immersion 24hrs before 4-OHT treatment (to prevent MMP production before immune cells are drawn to the epidermal pre-neoplastic cells) and subsequently for another 24hrs together with 4-OHT and imaged at 4dpf (Figure 3C). The same treatment was performed on the siblings for control tail fin wounding experiments as described above (Figure 3B). The same time frame of treatment was used for treatment with 200 μM Sivelestat (Tocris, 3535) and the phosphatase inhibitor mix consisting of: 100 μM (L-3- trans-carboxyoxirane-2-carbonyl)-l-leucine (3-methylbutyl) amide (E64c; Caymen Chemical), 0.04 TIU/ml Aprotinin (Tocris), 6 μM Leupeptin (Tocris) and 2 μM Pepstatin A (Tocris). Imaging and quantification of all the experiments were performed blinded.

#### Transmission electron microscopy/Scanning electron microscopy

Fish larvae were anaesthetised in 0.01 mg/ml tricaine, and embedded in 1% low melting point agarose bathed in 0.01 mg/ml tricaine in Danieau’s buffer after setting. For CLEM studies, larvae that had clones in the vicinity of the cloaca, which was selected as a morphological marker that can be seen pre and post processing, were selected and confocal images taken. After imaging, larvae were removed immediately from agarose and transferred to primary fix (4% glutaraldehyde, 1% paraformaldehyde, 0.05M sodium cacodylate, pH 7.4, 1 mM MgSO4, 1% sucrose) at 4°C overnight. Fixed samples were washed in 0.1M sodium cacodylate (3x10mins) and then secondary fixed in 2% osmium tetroxide, 0.1M sodium cacodylate at room temperature for 2 hours. After fixation, samples were washed 3x10 mins in 0.1M sodium cacodylate and then 2x10mins in dH_2_0 before serial dehydration in EtOH, 30 mins per EtOH concentration. Dehydration was completed by incubation in propylene oxide (PPO; 3 × 20 min). PPO was replaced with a 50:50 mix of PPO:epon, incubated overnight, and then evaporated off for 2 h. Samples were transferred twice to fresh epon (3 g TAAB 812 Resin, 2 g dodecenyl succinic anhydride, 1.25 g methyl nadic anhydride, and 0.1875 g benzyl dimethylamine) for 24 h and then embedded/polymerized at 60°C for 72 h. Sections were cut on an Ultramicrotome (Leica EM UC6) and imaged using a Tecnai 12-FEI 120-kV BioTwin Spirit Transmission Electron Microscope with a FEI Eagle 4k × 4k charge-coupled device camera. Manual image segmentation and false coloring was done using Adobe photoshop.

For SEM, samples were processed almost as for TEM except the fix mixtures: fix 1 was 2.5% glutaraldehyde, 0.1M sodium cacodylate and fix 2 was 1% osmium tetroxide, 0.1M sodium cacodylate. After EtOH dehydration samples were prepared using a Leica CPD300 critical point dryer, sputter coated with Au/Pd, using an Emitech 575X sputter coater, and examined in a FEI Quanta 200FEG SEM.

All *krt4:KalTA4-ER*^*t2*^ or *krt19:KalTA4-ER*^*t2*^ microinjected larvae were between 5 and 7dpf, Tg*(kita:Gal4;UAS:HRAS*^*G12V*^*-GFP)* larvae were 10dpf (Figure 2F) and 16dpf (Figure 2Aii)

#### Confocal imaging

Larvae were mounted on their sides in 1.0% low-melting agarose (Sigma), in a glass-bottomed dish, filled with Danieau’s buffer containing 0.01 mg/ml tricaine. Imaging was performed using a Leica TCS SP8 AOBS confocal laser scanning microscope attached to a Leica DMi8 inverted or a Leica DM6000 upright epifluorescence microscope using a 20x or 63x glycerol lens. Movies were exported from Volocity (PerkinElmer) as QuickTime movies using the Sorenson3 video compressor. For 3D reconstructions, imaging data were processed using either IMARIS software (Bitplane) or Volocity. Figures were prepared using Adobe Photoshop and Adobe Illustrator. Tracking of neutrophils was done using the Manual Tracking plugin from ImageJ.

#### EdU Labeling

Cell proliferation was assessed using the Click-iT Plus EdU Alexa Fluor 647 Imaging Kit (Life Technologies, C10640). Larvae were injected into the yolk with 0.5nl of 10mM EdU (5-ethynyl-2′-deoxyuridine, a nucleoside analog of thymidine) and incubated for 2.5 hours at 28.5°C. After a 30-min fixation with 4% paraformaldehyde (PFA) at room temperature (RT), larvae were permeabilized in PBS containing 0.5% Triton X-100 (PBST), washed and blocked with PBST containing 3% (w/v) Bovine Serum Albumin for 1 hour at RT. Larvae were then incubated with the Click-it Plus reaction cocktail containing Alexa Fluor picolyl azide 647 for 30 min at RT and later subjected to whole-mount immunofluorescence staining as described below. For eGFP immunostaining, larvae were washed in PBST 3 times for 15 min and re-blocked with PBST containing 5% (v/v) goat serum, 3% (w/v) Bovine Serum Albumin for 2 hours at room temperature, before an over-night incubation at 4°C with rabbit monoclonal anti-GFP antibody (1:200) (2956, Cell Signaling Technology). After 10 × 15-min PBST washes, larvae were incubated in Alexa Fluor 488 Goat anti-Rabbit secondary antibody (1:250) (A-11008, Invitrogen) for 2 hours at room temperature and washed again in PBST 10 times. Stained larvae were stored at 4°C in a glycerol based antifadent mountant (AF1, CitiFluor).

#### Immunofluorescent co-staining

5 dpf Tg(krt19:col 1α2-GFP) larvae were fixed with 2.5% PFA in PBS. After blocking with 5% goat serum in PBST, fixed larvae and primary antibodies to GFP and to collagen IV were incubated together (1:200). After extensive washing in multiple changes of PBST, larvae were incubated with Alexa Fluor 488 goat anti-mouse and Alexa Fluor 546 goat anti-rabbit secondary antibodies (1:500). After further PBST washes larvae were mounted in 1.5% agarose and imaged by confocal microscopy as above. Image analysis was performed using Fiji software.

### Quantification and Statistical Analysis

Statistical analyses were performed using Prism (GraphPad). Data were confirmed to be normally distributed via d’Agostino–Pearson test or Shapiro–Wilk test prior to further comparisons and an unpaired two-tailed Student’s t test or Wilcoxon-Mann-Whitney was used accordingly. Column scatter-plots show the mean ± SEM of all the individual data from repeated experiments or from a representative experiment as indicated in the figure legend. Significance values: ^∗^p ≤ 0.05, ^∗∗^p ≤ 0.001, ^∗∗∗^p ≤ 0.0001.

## Supplemental Information

Document S1. Figures S1–S4 and Table S1

Document S2. Article plus Supplemental Information

## References

[bib1] Antonio, N., Bonnelykke-Behrndtz, M.L., Ward, L.C., Collin, J., Christensen, I.J., Steiniche, T., Schmidt, H., Feng, Y., and Martin, P. (2015). The wound inflammatory response exacerbates growth of pre-neoplastic cells and progression to cancer. EMBO J. 34, 2219-2236. 10.15252/embj.201490147 PMC4585460 26136213

[bib2] Chang, T.T., Thakar, D., and Weaver, V.M., 2017. Force-dependent breaching of the basement membrane. Matrix Biol. 57-58, 178-189. 10.1016/j.matbio.2016.12.005 PMC5328923 28025167

[bib3] Chia, K., Mazzolini, J., Mione, M., and Sieger, D. (2018). Tumor initiating cells induce Cxcr4-mediated infiltration of pro-tumoral macrophages into the brain. eLife 7, e31918. 10.7554/eLife.31918 PMC5821457 29465400

[bib4] Coffelt, S.B., Kersten, K., Doornebal, C.W., Weiden, J., Vrijland, K., Hau, C.S., Verstegen, N.J.M., Ciampricotti, M., Hawinkels, L.J.A.C., Jonkers, J., and de Visser, K.E. (2015). IL-17-producing γδ T cells and neutrophils conspire to promote breast cancer metastasis. Nature 522, 345-348. 10.1038/nature14282 PMC4475637 25822788

[bib52] Distel, M., Wullimann, MF., Koster RW. Optimized Gal4 genetics for permanent gene expression mapping in zebrafish. 2009. PNAS Aug 11;106(32):13365-70. 10.1073/pnas.0903060106 PMC2726396 19628697

[bib5] Egeblad, M., Rasch, M.G., and Weaver, V.M. (2010). Dynamic interplay between the collagen scaffold and tumor evolution. Curr. Opin. Cell Biol. 22, 697-706. 10.1016/j.ceb.2010.08.015 PMC2948601 20822891

[bib6] Ellett, F., Pase, L., Hayman, J.W., Andrianopoulos, A., and Lieschke, G.J. (2011). mpeg1 promoter transgenes direct macrophage-lineage expression in zebrafish. Blood 117, e49-e56. 10.1182/blood-2010-10-314120 PMC3056479 21084707

[bib7] Feitosa, N.M., Richardson, R., Bloch, W., and Hammerschmidt, M. (2011). Basement membrane diseases in zebrafish. Methods Cell Biol. 105, 191-222. 10.1016/B978-0-12-381320-6.00008-4 21951531

[bib8] Feng, Y., Santoriello, C., Mione, M., Hurlstone, A., and Martin, P. (2010). Live imaging of innate immune cell sensing of transformed cells in zebrafish larvae: parallels between tumor initiation and wound inflammation. PLoS Biol. 8, e1000562. 10.1371/journal.pbio.1000562 PMC3001901 21179501

[bib9] Feng, Y., Renshaw, S., and Martin, P. (2012). Live imaging of tumor initiation in zebrafish larvae reveals a trophic role for leukocyte-derived PGE2. Curr. Biol. 22, 1253-1259. 10.1016/j.cub.2012.05.010 PMC3398414 22658594

[bib10] Fischer, B., Metzger, M., Richardson, R., Knyphausen, P., Ramezani, T., Franzen, R., Schmelzer, E., Bloch, W., Carney, T.J., and Hammerschmidt, M. (2014). p53 and TAp63 promote keratinocyte proliferation and differentiation in breeding tubercles of the zebrafish. PLoS Genet. 10, e1004048. 10.1371/journal.pgen.1004048 PMC3886889 24415949

[bib11] Frei, J.V. (1962). The fine structure of the basement membrane in epidermal tumors. J. Cell Biol. 15, 335-342. 10.1083/jcb.15.2.335 PMC2106146 13959516

[bib12] Freisinger, C.M., and Huttenlocher, A. (2014). Live imaging and gene expression analysis in zebrafish identifies a link between neutrophils and epithelial to mesenchymal transition. PLoS ONE 9, e112183. 10.1371/journal.pone.0112183 PMC4221567 25372289

[bib13] Gaggioli, C., Hooper, S., Hidalgo-Carcedo, C., Grosse, R., Marshall, J.F., Harrington, K., and Sahai, E. (2007). Fibroblast-led collective invasion of carcinoma cells with differing roles for RhoGTPases in leading and following cells. Nat. Cell Biol. 9, 1392-1400. 10.1038/ncb1658 18037882

[bib14] Glentis, A., Oertle, P., Mariani, P., Chikina, A., El Marjou, F., Attieh, Y., Zaccarini, F., Lae, M., Loew, D., Dingli, F., et al. (2017). Cancer-associated fibroblasts induce metalloprotease-independent cancer cell invasion of the basement membrane. Nat. Commun. 8, 924. 10.1038/s41467-017-00985-8 PMC5640679 29030636

[bib15] Gong, Z., Ju, B., Wang, X., He, J., Wan, H., Sudha, P.M., and Yan, T. (2002). Green fluorescent protein expression in germ-line transmitted transgenic zebrafish under a stratified epithelial promoter from keratin8. Dev. Dyn. 223, 204-215. 10.1002/dvdy.10051 11836785

[bib16] Hall, C., Flores, M.V., Storm, T., Crosier, K., and Crosier, P. (2007). The zebrafish lysozyme C promoter drives myeloid-specific expression in transgenic fish. BMC Dev. Biol. 7, 42. 10.1186/1471-213X-7-42 PMC1877083 17477879

[bib17] Hall, C.J., Boyle, R.H., Sun, X., Wicker, S.M., Misa, J.P., Krissansen, G.W., Print, C.G., Crosier, K.E., and Crosier, P.S. (2014). Epidermal cells help coordinate leukocyte migration during inflammation through fatty acid-fuelled matrix metalloproteinase production. Nat. Commun. 5, 3880. 10.1038/ncomms4880 24852213

[bib18] He, S., Lamers, G.E., Beenakker, J.-W.M., Cui, C., Ghotra, V.P., Danen, E.H., Meijer, A.H., Spaink, H.P., and Snaar-Jagalska, B.E. (2012). Neutrophil-mediated experimental metastasis is enhanced by VEGFR inhibition in a zebrafish xenograft model. J. Pathol. 227, 431-445. 10.1002/path.4013 PMC3504093 22374800

[bib19] Howat, W.J., Holmes, J.A., Holgate, S.T., and Lackie, P.M. (2001). Basement membrane pores in human bronchial epithelium: a conduit for infiltrating cells? Am. J. Pathol. 158, 673-680. 10.1016/S0002-9440(10)64009-6 PMC1850329 11159204

[bib20] Hynes, R.O. (2012). The evolution of metazoan extracellular matrix. J. Cell Biol. 196, 671-679. 10.1083/jcb.201109041 PMC3308698 22431747

[bib21] Imboden, M., Goblet, C., Korn, H., and Vriz, S. (1997). Cytokeratin 8 is a suitable epidermal marker during zebrafish development. C. R. Acad. Sci. III 320, 689-700. 10.1016/s0764-4469(97)84816-0 9377174

[bib22] Kajita, M., Hogan, C., Harris, A.R., Dupre-Crochet, S., Itasaki, N., Kawakami, K., Charras, G., Tada, M., and Fujita, Y. (2010). Interaction with surrounding normal epithelial cells influences signaling pathways and behavior of Src-transformed cells. J. Cell Sci. 123, 171-180. 10.1242/jcs.057976 PMC2954245 20026643

[bib23] Kinjo, M. (1978). Lodgement and extravasation of tumour cells in blood-borne metastasis: an electron microscope study. Br. J. Cancer 38, 293-301. 10.1038/bjc.1978.201 PMC2009708 698045

[bib24] Kitamura, T., Qian, B.Z., and Pollard, J.W. (2015a). Immune cell promotion of metastasis. Nat. Rev. Immunol. 15, 73-86. 10.1038/nri3789 PMC4470277 25614318

[bib25] Kitamura, T., Qian, B.Z., Soong, D., Cassetta, L., Noy, R., Sugano, G., Kato, Y., Li, J., and Pollard, J.W. (2015b). CCL2-induced chemokine cascade promotes breast cancer metastasis by enhancing retention of metastasis-associated macrophages. J. Exp. Med. 212, 1043-1059. 10.1084/jem.20141836 PMC4493415 26056232

[bib26] Krall, J.A., Reinhardt, F., Mercury, O.A., Pattabiraman, D.R., Brooks, M.W., Dougan, M., Lambert, A.W., Bierie, B., Ploegh, H.L., Dougan, S.K., and Weinberg, R.A. (2018). The systemic response to surgery triggers the outgrowth of distant immune-controlled tumors in mouse models of dormancy. Sci. Transl. Med. 10, eaan3464. 10.1126/scitranslmed.aan3464 PMC6364295 29643230

[bib27] Kwan, K.M., Fujimoto, E., Grabher, C., Mangum, B.D., Hardy, M.E., Campbell, D.S., Parant, J.M., Yost, H.J., Kanki, J.P., and Chien, C.B. (2007). The Tol2kit: a multisite gateway-based construction kit for TOl2 transposon transgenesis constructs. Dev. Dyn. 236, 3088-3099. 10.1002/dvdy.21343 17937395

[bib28] Le Guellec, D., Morvan-Dubois, G., and Sire, J.Y. (2004). Skin development in bony fish with particular emphasis on collagen deposition in the dermis of the zebrafish (Danio rerio). Int. J. Dev. Biol. 48, 217-231. 10.1387/ijdb.15272388 15272388

[bib29] Lee, R.T.H., Asharani, P.V., and Carney, T.J. (2014). Basal keratinocytes contribute to all strata of the adult zebrafish epidermis. PLoS ONE 9, e84858. 10.1371/journal.pone.0084858 PMC3882266 24400120

[bib30] Li, Q., Frank, M., Thisse, C.I., Thisse, B.V., and Uitto, J. (2011). Zebrafish: a model system to study heritable skin diseases. J. Invest. Dermatol. 131, 565-571. 10.1038/jid.2010.388 PMC3342776 21191402

[bib31] Liongue, C., Hall, C.J., O’Connell, B.A., Crosier, P., and Ward, A.C. (2009). Zebrafish granulocyte colony-stimulating factor receptor signaling promotes myelopoiesis and myeloid cell migration. Blood 113, 2535-2546. 10.1182/blood-2008-07-171967 19139076

[bib32] McAlindon, M.E., Gray, T., Galvin, A., Sewell, H.F., Podolsky, D.K., and Mahida, Y.R. (1998). Differential lamina propria cell migration via basement membrane pores of inflammatory bowel disease mucosa. Gastroenterology 115, 841-848. 10.1016/s0016-5085(98)70255-0 9753486

[bib33] Menter, D.G., and Dubois, R.N. (2012). Prostaglandins in cancer cell adhesion, migration, and invasion. Int. J. Cell Biol. 2012, 723419. 10.1155/2012/723419 PMC3299390 22505934

[bib34] Morris, J.L., Cross, S.J., Lu, Y., Kadler, K.E., Lu, Y., Dallas, S.L., and Martin, P. (2018). Live imaging of collagen deposition during skin development and repair in a collagen I - GFP fusion transgenic zebrafish line. Dev. Biol. 441, 4-11. 10.1016/j.ydbio.2018.06.001 PMC6080847 29883658

[bib35] Nauroy, P., Hughes, S., Naba, A., and Ruggiero, F. (2018). The in-silico zebrafish matrisome: A new tool to study extracellular matrix gene and protein functions. Matrix Biol. 65, 5-13. 10.1016/j.matbio.2017.07.001 28739138

[bib36] Nauroy, P., Guiraud, A., Chlasta, J., Malbouyres, M., Gillet, B., Hughes, S., Lambert, E., and Ruggiero, F. (2019). Gene profile of zebrafish fin regeneration offers clues to kinetics, organization and biomechanics of basement membrane. Matrix Biol. 75-76, 82-101. 10.1016/j.matbio.2018.07.005 30031067

[bib37] Ramezani, T., Laux, D.W., Bravo, I.R., Tada, M., and Feng, Y. (2015). Live imaging of innate immune and preneoplastic cell interactions using an inducible Gal4/UAS expression system in larval zebrafish skin. J. Vis. Exp. 96, e52107. 10.3791/52107 PMC4354608 25741625

[bib38] Renkawitz, J., Kopf, A., Stopp, J., de Vries, I., Driscoll, M.K., Merrin, J., Hauschild, R., Welf, E.S., Danuser, G., Fiolka, R., and Sixt, M. (2019). Nuclear positioning facilitates amoeboid migration along the path of least resistance. Nature 568, 546-550. 10.1038/s41586-019-1087-5 PMC7217284 30944468

[bib39] Rhodes, J., Hagen, A., Hsu, K., Deng, M., Liu, T.X., Look, A.T., and Kanki, J.P. (2005). Interplay of pu.1 and gata1 determines myelo-erythroid progenitor cell fate in zebrafish. Dev. Cell 8, 97-108. 10.1016/j.devcel.2004.11.014 15621533

[bib40] Sabeh, F., Shimizu-Hirota, R., and Weiss, S.J. (2009). Protease-dependent versus -independent cancer cell invasion programs: three-dimensional amoeboid movement revisited. J. Cell Biol. 185, 11-19. 10.1083/jcb.200807195 PMC2700505 19332889

[bib41] Santoriello, C., Gennaro, E., Anelli, V., Distel, M., Kelly, A., Koster, R.W., Hurlstone, A., and Mione, M. (2010). Kita driven expression of oncogenic HRAS leads to early onset and highly penetrant melanoma in zebrafish. PLoS ONE 5, e15170. 10.1371/journal.pone.0015170 PMC3000817 21170325

[bib42] Schafer, M., and Werner, S. (2008). Cancer as an overhealing wound: an old hypothesis revisited. Nat. Rev. Mol. Cell Biol. 9, 628-638. 10.1038/nrm2455 18628784

[bib43] Sherwood, D.R., and Sternberg, P.W. (2003). Anchor cell invasion into the vulval epithelium in C. elegans. Dev. Cell 5, 21-31. 10.1016/s1534-5807(03)00168-0 12852849

[bib44] Spaderna, S., Schmalhofer, O., Hlubek, F., Berx, G., Eger, A., Merkel, S., Jung, A., Kirchner, T., and Brabletz, T. (2006). A transient, EMT-linked loss of basement membranes indicates metastasis and poor survival in colorectal cancer. Gastroenterology 131, 830-840. 10.1053/j.gastro.2006.06.016 16952552

[bib45] Spenle, C., Hussenet, T., Lacroute, J., Lefebvre, O., Kedinger, M., Orend, G., and Simon-Assmann, P. (2012). Dysregulation of laminins in intestinal inflammation. Pathol. Biol. (Paris) 60, 41-47. 10.1016/j.patbio.2011.10.005 22100883

[bib46] Szalayova, G., Ogrodnik, A., Spencer, B., Wade, J., Bunn, J., Ambaye, A., James, T., and Rincon, M. (2016). Human breast cancer biopsies induce eosinophil recruitment and enhance adjacent cancer cell proliferation. Breast Cancer Res. Treat. 157, 461-474. 10.1007/s10549-016-3839-3 PMC5026505 27249999

[bib47] Takeuchi, T., and Gonda, T. (2004). Distribution of the pores of epithelial basement membrane in the rat small intestine. J. Vet. Med. Sci. 66, 695-700. 10.1292/jvms.66.695 15240945

[bib48] Travnickova, J., Tran Chau, V., Julien, E., Mateos-Langerak, J., Gonzalez, C., Lelievre, E., Lutfalla, G., Tavian, M., and Kissa, K. (2015). Primitive macrophages control HSPC mobilization and definitive haematopoiesis. Nat. Commun. 6, 6227. 10.1038/ncomms7227 25686881

[bib49] Voisin, M.B., Probstl, D., and Nourshargh, S. (2010). Venular basement membranes ubiquitously express matrix protein low-expression regions: characterization in multiple tissues and remodeling during inflammation. Am. J. Pathol. 176, 482-495. 10.2353/ajpath.2010.090510 PMC2797906 20008148

[bib50] Westerfield, M. (2007). The Zebrafish Book: A Guide for the Laboratory Use of Zebrafish (Danio Rerio), 5th Edition (University of Oregon Press).

[bib51] Wolf, K., Muller, R., Borgmann, S., Brocker, E.B., and Friedl, P. (2003). Amoeboid shape change and contact guidance: T-lymphocyte crawling through fibrillar collagen is independent of matrix remodeling by MMPs and other proteases. Blood 102, 3262-3269. 10.1182/blood-2002-12-3791 12855577

