# Supplementary material for: Proteolytic and Opportunistic Breaching of the Basement Membrane Zone by Immune Cells during Tumor Initiation
Source: Cell Rep. 2019 Jun 4;27(10):2837–2846.e4. doi: 10.1016/j.celrep.2019.05.029 (PMC6581915; doi:10.1016/j.celrep.2019.05.029)

Cell Reports, Volume *27*

Supplemental Information

Proteolytic and Opportunistic Breaching of the Basement Membrane Zone by Immune Cells during Tumor Initiation

Maaike C.W. van den Berg, Lucy MacCarthy-Morrogh, Deborah Carter, Josephine Morris, Isabel Ribeiro Bravo, Yi Feng, and Paul Martin

Supplemental Figure legends

Figure S1. Basal pre-neoplastic epidermal cells recruit neutrophils and macrophages. Related to Figure 1

Increased neutrophil (magenta) (n=38 and 15) and macrophage (red) (n= 35 and 50) recruitment to $HRAS^{G12V}$-expressing pre-neoplastic basal cells (green) compared to control GFP-CAAX-expressing basal cells, quantified on the right. Scale Bars: 100µm. Graphs display mean ± SEM.

Figure S2. Protease or elastase inhibition does not affect neutrophil or macrophage recruitment to pre-neoplastic superficial cells. Related to Figure 3

A. Protease inhibitor mix (PI mix) does not affect the number of neutrophils (magenta n=28 and n=24) or macrophages (red n=26 and n=30) recruited to pre-neoplastic superficial cells. **B.** Neutrophil elastase (Sivelestat) treatment does not affect neutrophil (magenta n=18 and n=21) or macrophage (red n=15 and n=20) recruitment to pre-neoplastic superficial cells.

Neutrophils and macrophages reside along myosepta in wild type larvae. **C**. Diagram shows localisation of immune cells along horizontal myoseptum (midline) of the flank of the fish. Below are images of neutrophils (magenta) and macrophages (red) residing along the horizontal myoseptum in 5dpf larva. **D.** 14dpf larva (internal view) showing macrophages (red) migrating along myosepta (arrowheads). **E**. Older larva (14dpf) showing gaps in collagen I layer (gray) along vertical myoseptum (arrowheads).

Scale Bars: (C, E) 20µm, (A, B and D) 50µm. Graphs display mean ± SEM.

Figure S3. Pre-neoplastic superficial cells have taken up collagen I-GFP. Related to Figure 4

A. 2 single z-stacks from image in Fig. 4E/F to indicate collagen uptake (green) by pre-neoplastic cells (red) (white arrows). **B.** Different pre-neoplastic superficial clone showing collagen inside pre-neoplastic cell (asterisks) in single z-stack (Bi) and in 3D projection (Bii). **C.** Breach (red arrowheads in **Cii**) in BM (false coloured in green in **Ci**) underneath superficial pre-neoplastic clone above horizontal myoseptum.

Scale bars: (A) 20μm, (B) 5μm, (Ci) 10μm, (Cii) 1μm

Figure S4. Schematic depicting how immune cells might traverse the BMZ barrier layer to access pre-neoplastic skin cells. Related to Figure 4. Along the horizontal myoseptum pre-existing gaps in the basement membrane zone can be used by immune cells to access the epidermis (**A**). It is in these areas that pre-neoplastic clones preferentially grow (**B**). Once the pre-neoplastic cells start growing, additional holes appear in the vicinity which immune cells can also use to access the epidermis (**C**). Finally, skin wounding, for example due to biopsy/surgery, creates a larger portal which also allows immune cells access to the epidermis (**D**).

Table S1. Transgenic zebrafish lines. Related to STAR METHODS

**Transgenic zebrafish lines**	**Reference**
Tg*(krt4:GFP)*	(Gong et al., 2002)
Tg*(krt19:tdTomatoCAAX)*	(Lee et al., 2014), (Morris et al., 2018)
Tg(*lyz:DsRed*)	(Hall et al., 2007)
Tg*(mpeg1:mCherry)*	(Ellett et al., 2011)
Tg(*UAS:GAP43-GFP*)	(Kajita et al., 2010)
Tg(*UAS:eGFP*)	(Santoriello et al., 2010)
Tg*(6xUAS:mCherry-HRAS^{G12V})*	This manuscript
Tg*(5XUAS:eGFP-HRAS^{V12})*	(Santoriello et al., 2010)
Et(*kita:GalTA4, UAS:mcherry*)	(Distel et al., 2009)
Tg*(kita:Gal4;UAS:HRAS^{G12V}-GFP)*	(Santoriello et al., 2010),(Feng et al., 2010)
Tg(*krt19:col1α2-GFP*)	(Morris et al., 2018)
Tg*(krt19:col1α2-GFP;lyz:dsRed)*	This manuscript
Tg(*krt19:col1α2-GFP*;*mpeg1:mcherry)*	This manuscript
Tg*(krt19:col1α2-GFP;UAS:mCherry-HRAS^{G12V})*	This manuscript
Tg*(krt19:col1α2-GFP;mpeg1:mCherry; UAS:mCherry-HRAS^{G12V})*	This manuscript
Tg*(krt19:col1α2-GFP; lyz:dsRed;UAS:mCherry-HRAS^{G12V})*	This manuscript

FIGURE S1

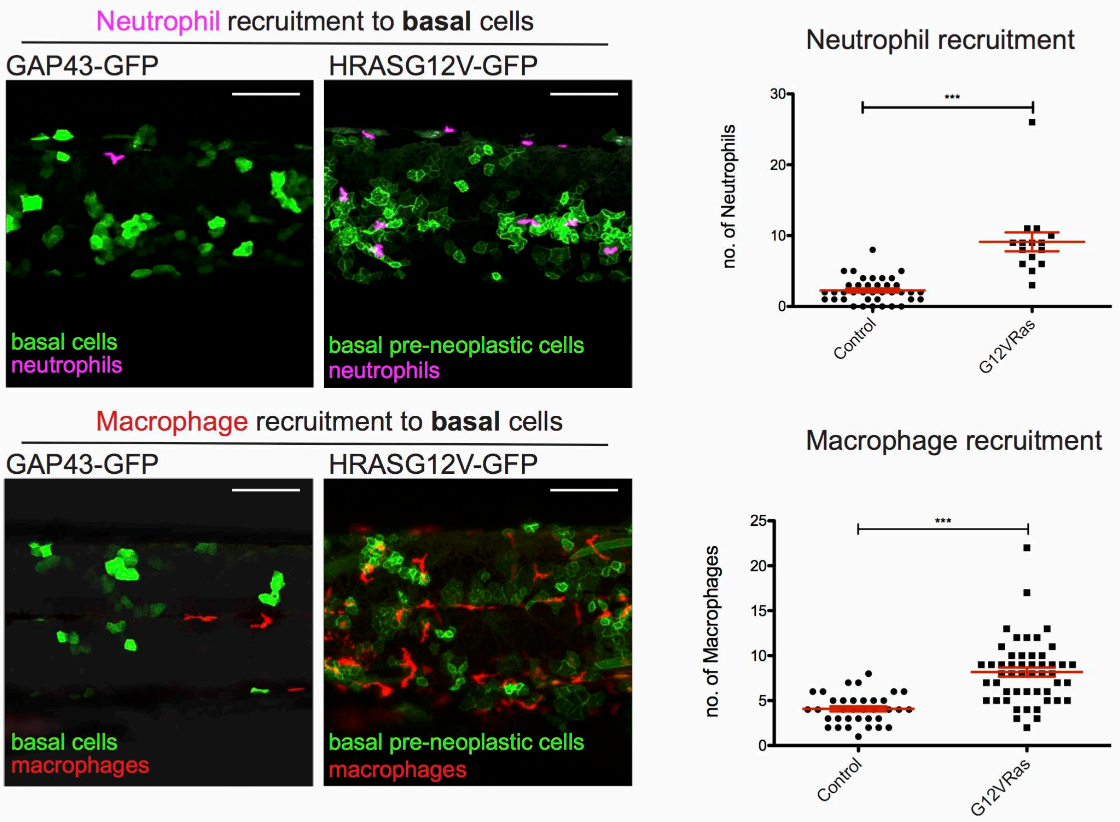

Neutrophil recruitment to basal cells
GAP43-GFP
HRASG12V-GFP
basal cells
neutrophils
basal pre-neoplastic cells
neutrophils
Macrophage recruitment to basal cells
GAP43-GFP
HRASG12V-GFP
basal cells
macrophages
basal pre-neoplastic cells
macrophages
Neutrophil recruitment
no. of Neutrophils
30
20
10
0
***
Control
G12VRas
Macrophage recruitment
no. of Macrophages
25
20
15
10
5
0
***
Control
G12VRas

FIGURE S2

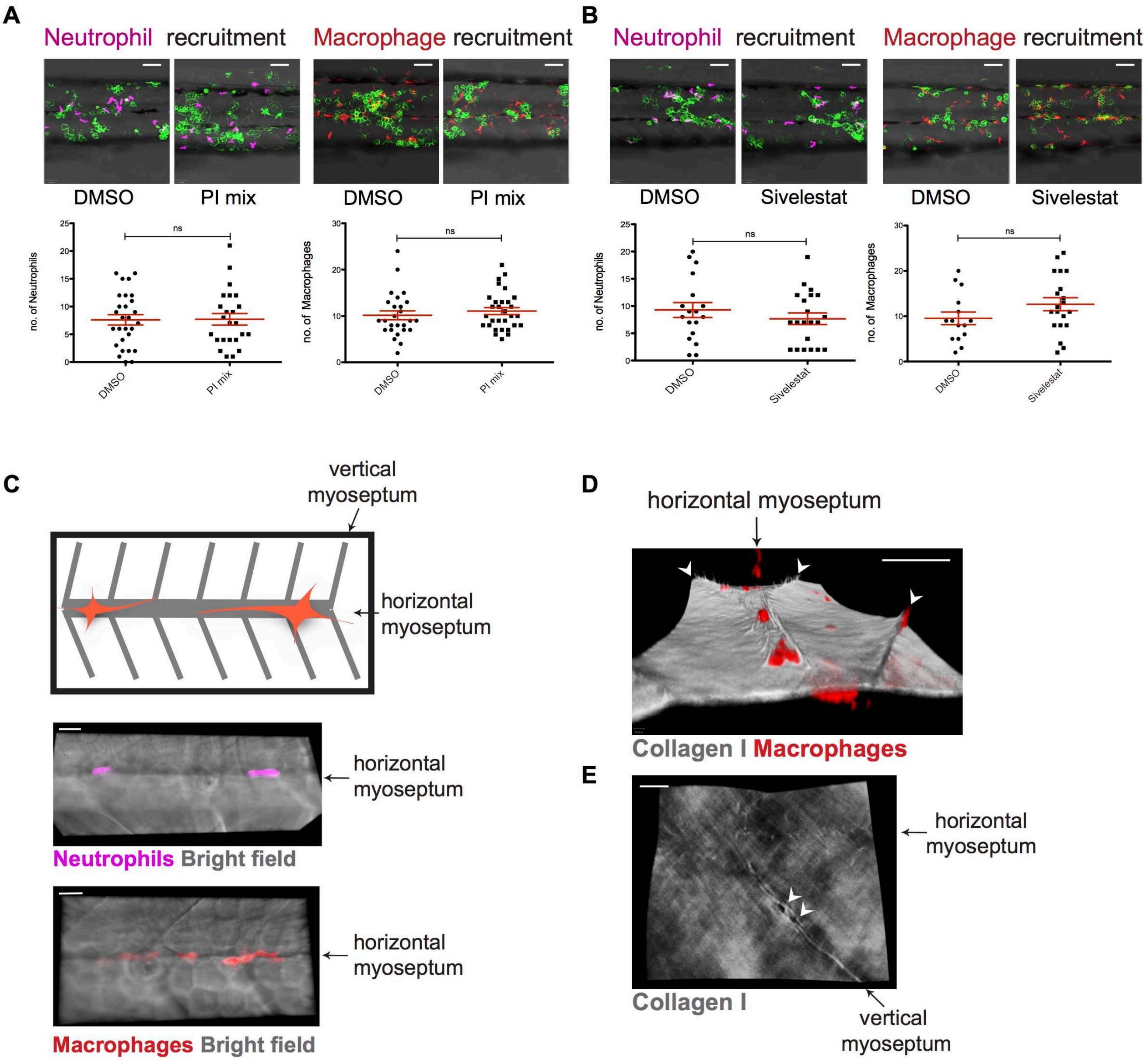
A
Neutrophil recruitment
Macrophage recruitment
DMSO
PI mix
DMSO
PI mix
no. of Neutrophils
ns
DMSO
PI mix
no. of Macrophages
ns
DMSO
PI mix
B
Neutrophil recruitment
Macrophage recruitment
DMSO
Sivelestat
DMSO
Sivelestat
no. of Neutrophils
ns
DMSO
Sivelestat
no. of Macrophages
ns
DMSO
Sivelestat
C
vertical myoseptum
horizontal myoseptum
horizontal myoseptum
Neutrophils Bright field
horizontal myoseptum
Macrophages Bright field
D
horizontal myoseptum
Collagen I Macrophages
E
horizontal myoseptum
Collagen I
vertical myoseptum

FIGURE S3

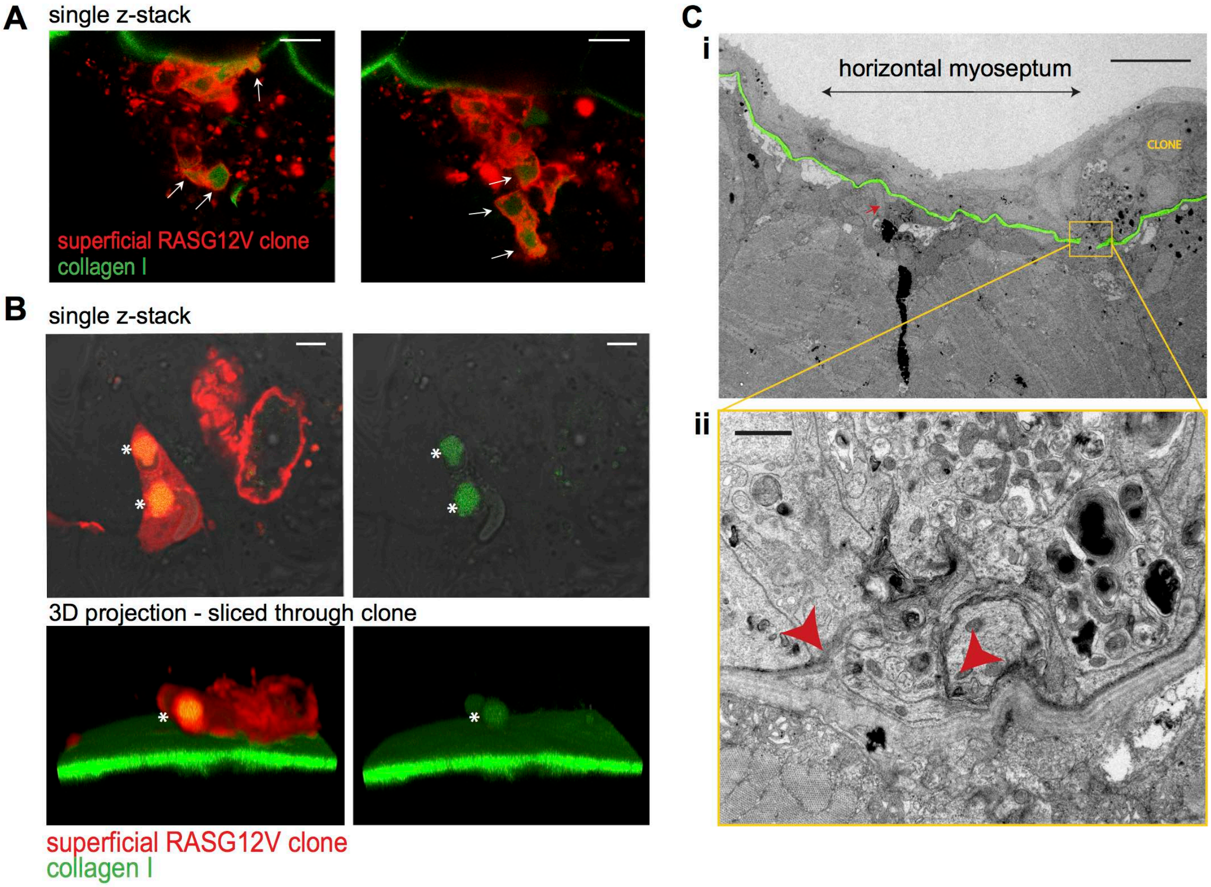
A
single z-stack
superficial RASG12V clone
collagen I
B
single z-stack
3D projection - sliced through clone
superficial RASG12V clone
collagen I
C
i
horizontal myoseptum
CLONE
ii

FIGURE S4

A. Innate immune cells make use of opportunistic holes in collagen I layer to access the skin in healthy larvae

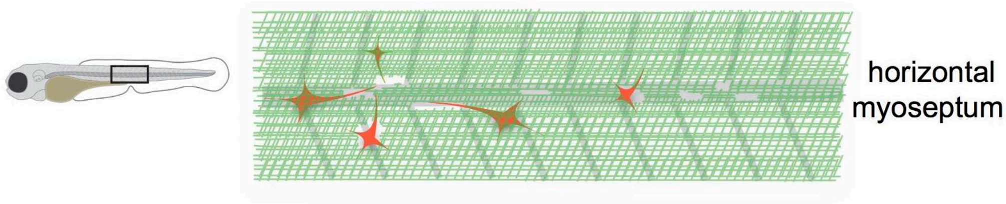

B. Cancer clones preferentially grow along the midline where there is easier access for immune cells to contact the pre-neoplastic cells

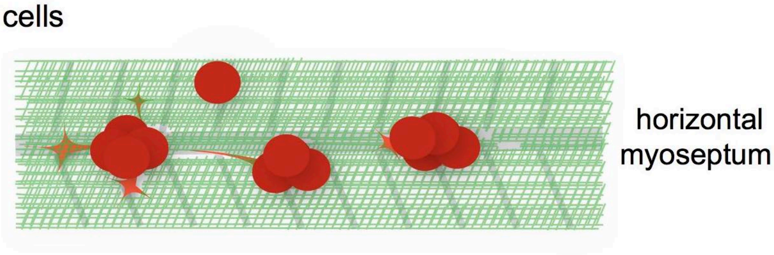

C. In the presence of larger cancer clones additional holes appear that are used by immune cells to access the clone

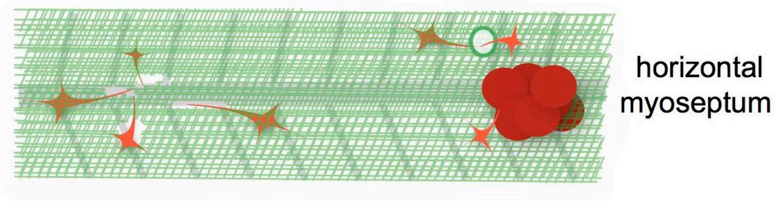

D. Mechanical breaching/wounding of the basement membrane zone results in a larger portal for immune cells to gain access to the skin

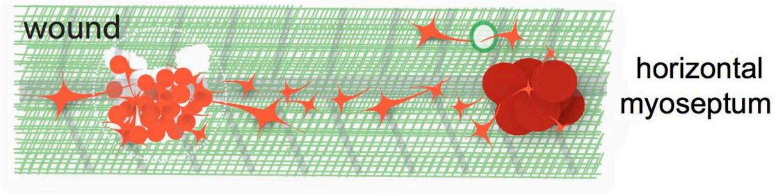

Supplement: Document S1. Figures S1–S4 and Table S1 [file mmc1.pdf]
